# Editorial: Microbial response to emerging contaminants in soil and sediment ecosystems

**DOI:** 10.3389/fmicb.2024.1371223

**Published:** 2024-02-13

**Authors:** Cheng Zhang, Aiju Liu, Mezbaul Bahar

**Affiliations:** ^1^School of Environment & Ecology, Jiangnan University, Wuxi, China; ^2^School of Resources and Environmental Engineering, Shandong University of Technology, Zibo, China; ^3^Global Centre for Environmental Remediation, The University of Newcastle, Callaghan, NSW, Australia

**Keywords:** microorganisms, microplastic, meta-analysis, soil microbial community, microbial function

Emerging contaminants include persistent organic pollutants, endocrine disruptors (ECDs), microplastics, and antibiotics (Puri et al., 2023). These emerging contaminants are increasingly seeping into our soil and sediment ecosystems, causing increasing environmental problems for ecosystems. Besides, nanomaterials are often used in environmental remediation processes, but their entry into the environment may have toxic effects on microorganisms, such as death or cell damage of microorganisms, as well as genetic mutations or variations of microorganisms (Wang et al., 2024). Since the large-scale production of plastic products, microplastics have accumulated in the soil, water, and other environments (Kane et al., 2020). Due to a variety of sources such as irrigation, fertilization, sludge application, and atmospheric sedimentation, the soil system has become an important sink (Tian et al., 2022). To assess whether plastics cause different responses to soil microbiome parameters, a meta-analysis of published literature was conducted to quantitatively assess the effects of plastics on soil microbiome biomass, diversity, and function.

This Research Topic includes six different articles. In particular, Li et al. found that plastics accelerated the loss of soil organic carbon and significantly increased the function of microorganisms. Plastics also affected the soil microbial community by changing the physical and chemical properties of soil and reducing the stability of microbial biomass and co-occurrence network. In addition to microplastics, antibiotics, and heavy metals, have also attracted wide attention for their environmental toxic effects and pollution mechanisms on soil in recent years, especially the comprehensive toxic effects of these two substances on soil microorganisms (Wang et al., 2021). As the backbone of various environmental ecosystems, microbial communities play a key role in maintaining ecological balance and function. There is therefore a need to understand how these contaminants alter microbial diversity, function, and the overall health of soil and sediment environments.

Ammonia-oxidizing archaea and bacteria and their related soil nitrification functions play a key role in soil nitrogen turnover. Therefore, it is widely used to assess the ecological risks of soil pollution (Chen et al., 2023). Hou et al. found that the impacts of sulfadiazine (SDZ) and copper (Cu) on ammonia-oxidizing archaea and ammonia-oxidizing bacteria communities depended on soil types, and the specific phylotype corresponded to the PNR variation under the stress of SDZ and Cu. Microorganisms play a key role in the soil biogeochemical cycle, and in addition to the effects of emerging contaminants released into the environment, changes in the microbial community structure in the soil also interact with the characteristics of the soil environment (Du et al., 2022). Liu J. et al. found that soil enzyme activity and potential nitrification rate largely depended on the composition and richness of the bacterial community rather than the diversity, which were greatly changed by the availability of soil nitrogen and phosphorus. According to Symochko et al. cellulose-destroying microorganisms are chemical rot bacteria with high organic matter content in soil, and intensive agricultural practices in Ukrainian soil have greatly changed the content and composition of organic matter, resulting in a decrease in humus and soil organic matter reserves.

Soil is responsible for many productive activities, such as mining and construction, but these activities affect the community structure and function of soil microorganisms to varying degrees. Coal mining is an important production activity, and it is also the most obvious example of man-made degradation of the ecological environment (Liu et al., 2016). It is very important to choose the appropriate vegetation restoration method to maintain the stability of the artificial restoration soil ecosystem in mining areas (Li and Liber, 2018). Liu S. et al. found that the bacterial community structure and diversity were closely related to the age and type of forest restoration, and soil total carbon, total nitrogen, ${\text{NH}}_{4}^{+}$-N, total phosphorus, and pH were the main environmental factors to the bacterial community structure of coniferous forest and broadleaf forest, and this study was crucial to promote the use of microorganisms to reveal the effect of environmental restoration. Promoting energy transformation is crucial to the realization of China's dual-carbon goal, and the use of clean energy such as solar energy and wind energy has helped this transformation to a certain extent. However, the use of these two kinds of energy cannot be separated from the construction of solar panels and windmills. Now, many scholars are studying the impact of solar panel construction on the environment. At present, there is a view that the installation of solar power plants is considered a “win-win” strategy because it can reduce carbon emissions and prevent desertification in arid areas at the same time (Liu et al., 2020). Liu Z. et al. assessed the potential environmental benefits and risks of solar photovoltaic power plants from the perspective of soil microbial ecosystems and found that solar photovoltaic devices affected plant aboveground biomass to change the total nitrogen content. In addition to the direct impact, changes in plant aboveground biomass also indirectly drove the diversity of fungal communities by changing the total nitrogen content in the soil. Reducing the proportion of functional microbiota, the temperature drop caused by solar panel shading also directly inhibited the growth and activity of soil microorganisms (Yue et al., 2021).

In general, microorganisms play an important role in soil biogeochemical cycles. This Research Topic aims to explore and continue to pay attention to the impact that emerging contaminants (such as microplastics and heavy metals) on microorganisms and ecosystems in soil and sediment ecosystems. And stimulate the readership's interest in the various effects of emerging contaminants in soil ecosystems.

## Author contributions

CZ: Conceptualization, Writing – original draft. AL: Supervision, Writing – review & editing. MB: Supervision, Writing – review & editing.

## References

[B1] Chen J. Zhao S. Gan Y. T. Wu J. Dai J. C. Chao H. J.. (2023). Dichlorodiphenyltrichloroethane inhibits soil ammonia oxidation by altering ammonia-oxidizing archaeal and bacterial communities. Environ. Pollut. 333, 122063. 10.1016/j.envpol.2023.122063 37330184

[B2] Du M. Zheng M. G. Liu A. F. Wang L. Pan X. Liu J.. (2022). Effects of emerging contaminants and heavy metals on variation in bacterial communities in estuarine sediments. Sci. Total Environ. 832, 155118. 10.1016/j.scitotenv.2022.155118 35398136

[B3] Kane I. A. Clare M. A. Miramontes E. Wogelius R. Rothwell J. J. Garreau P.. (2020). Seafloor microplastic hotspots controlled by deep-sea circulation. Science 368, 1140–1145. 10.1126/science.aba5899 32354839

[B4] Li S. Q. Liber K. (2018). Influence of different revegetation choices on plant community and soil development nine years after initial planting on a reclaimed coal gob pile in the Shanxi mining area, China. Sci. Total Environ. 618, 1314–1323. 10.1016/j.scitotenv.2017.09.252 29046231

[B5] Liu X. Y. Zhou W. Bai Z. K. (2016). Vegetation coverage change and stability in large open-pit coal mine dumps in China during 1990–2015. Ecol. Eng. 95, 447–451. 10.1016/j.ecoleng.2016.06.051

[B6] Liu Y. Zhang R. Q. Ma X. R. Wu G. L. (2020). Combined ecological and economic benefits of the solar photovoltaic industry in arid sandy ecosystems. J. Clean. Prod. 262, 121376. 10.1016/j.jclepro.2020.121376

[B7] Puri M. Gandhi K. Kumar M. S. (2023). Emerging environmental contaminants: a global perspective on policies and regulations. J. Environ. Manage. 332, 117344. 10.1016/j.jenvman.2023.117344 36736081

[B8] Tian L. Jinjin C. Ji R. Ma Y. Yu X. (2022). Microplastics in agricultural soils: sources, effects, and their fate. Curr. Opin. Environ. Sci. Health 25, 100311. 10.1016/j.coesh.2021.100311

[B9] Wang C. Q. Chen L. A. Xu J. K. Zhang L. L. Yang X. Q. Zhang X. K.. (2024). Environmental behaviors and toxic mechanisms of engineered nanomaterials in soil. Environ. Res. 242, 117820. 10.1016/j.envres.2023.117820 38048867

[B10] Wang J. Peng C. Li H. Zhang P. Liu X. (2021). The impact of microplastic-microbe interactions on animal health and biogeochemical cycles: a mini-review. Sci. Total Environ. 773, 145697. 10.1016/j.scitotenv.2021.145697 33940764

[B11] Yue S. Guo M. Zou P. Wu W. Zhou X. (2021). Effects of photovoltaic panelson soil temperature and moisture in desert areas. Environ. Sci. Pollut. Res. 28, 17506–17518. 10.1007/s11356-020-11742-8 33400111

